# Variational based smoke removal in laparoscopic images

**DOI:** 10.1186/s12938-018-0590-5

**Published:** 2018-10-19

**Authors:** Congcong Wang, Faouzi Alaya Cheikh, Mounir Kaaniche, Azeddine Beghdadi, Ole Jacob Elle

**Affiliations:** 1 Norwegian Colour and Visual Computing Lab, Norwegian University of Science and Technology, Gjøvik, Norway; 2 L2TI-Institut Galilée, Université Paris 13, Sorbonne Paris Cité, Villetaneuse, France; 3 The Intervention Centre, Oslo University Hospital, Oslo, Norway; 4 The Department of Informatics, University of Oslo, Oslo, Norway

**Keywords:** Laparoscopic images, Smoke removal, Dehazing, Variational approach, Image quality

## Abstract

**Background:**

In laparoscopic surgery, image quality can be severely degraded by surgical smoke, which not only introduces errors for the image processing algorithms (used in image guided surgery), but also reduces the visibility of the observed organs and tissues. To overcome these drawbacks, this work aims to remove smoke in laparoscopic images using an image preprocessing method based on a variational approach.

**Methods:**

In this paper, we present the physical smoke model where the degraded image is separated into two parts: *direct attenuation* and *smoke veil* and propose an efficient variational-based desmoking method for laparoscopic images. To estimate the *smoke veil*, the proposed method relies on the observation that *smoke veil* has low contrast and low inter-channel differences. A cost function is defined based on this prior knowledge and is solved using an augmented Lagrangian method. The obtained *smoke veil* is then subtracted from the original degraded image, resulting in the *direct attenuation* part. Finally, the smoke free image is computed using a linear intensity transformation of the *direct attenuation* part.

**Results:**

The performance of the proposed method is evaluated quantitatively and qualitatively using three datasets: two public real smoked laparoscopic datasets and one generated synthetic dataset. No-reference and reduced-reference image quality assessment metrics are used with the two real datasets, and show that the proposed method outperforms the state-of-the-art ones. Besides, standard full-reference ones are employed with the *synthetic dataset*, and indicate also the good performance of the proposed method. Furthermore, the qualitative visual inspection of the results shows that our method removes smoke effectively from the laparoscopic images.

**Conclusion:**

All the obtained results show that the proposed approach reduces the smoke effectively while preserving the important perceptual information of the image. This allows to provide a better visualization of the operation field for surgeons and improve the image guided laparoscopic surgery procedure.

## Background

In the last decade, with the technological advances in laparoscopic devices, medical imaging and the demand for minimally invasive approaches have led to an increase in the number of laparoscopic surgeries [1]. During a laparoscopic surgery, a number of small incisions are made by the oncologist, then a needle is inserted to expand the abdomen with carbon dioxide gas to allow room for other instruments. The oncologist uses specialized instruments for example telescope, ultrasonic probe to visualize the abdominal cavity. Thus the video/images captured by the laparoscope is one of the most important intra-operative data modality. A high video/images quality is of vital importance for the operating surgeons and for computer vision based navigation systems [2–4].

However, the artifacts during laparoscopic surgery which include smoke, blood, dynamic illumination conditions, specular reflections, etc., [5] deteriorate image quality. In particular, smoke caused by such as laser ablation and electrocautery [6] significantly reduces the contrast and radiance information for large areas of the scene. Surgeons’ visibility would inevitably suffer from this degradation [2]. Besides, computer vision algorithms developed in image guided navigation systems are mainly for clear images, smoke would influence their performance especially in heavy smoke area [7]. Therefore, when smoke is detected [8], smoke removal by image processing techniques in laparoscopic surgery becomes necessary to provide a clear operation field visualization for surgeons and to avoid degradation of the performance of computer vision algorithms.

In this paper, we present the physical smoke model and propose an efficient variational based desmoking method for laparoscopic images. Instead of using the widely employed atmospheric scattering model [9, 10] and estimating the transmission or depth map as well as the atmospheric light, we propose to resort to a smoke model where the degraded image is separated into two parts: *direct attenuation* and *smoke veil*. The estimation of the *smoke veil* relies on two assumptions: *smoke veil* has low contrast and low RGB inter-channel differences. Then the *direct attenuation* part is obtained by subtracting the *smoke veil* from original degraded image. Finally, the smoke free image is recovered from the *direct attenuation* part by linear transformation of intensity.

The remainder of this paper is organized as follows. In  “Related work” section, a review of laparoscopic image desmoking methods as well as image dehazing is given. In “Physical model of smoke image acquisition” section, the retained physical model for smoke image is derived from the atmospheric scattering model. “Proposed smoke removal approach” section describes our proposed approach by defining the energy function and the optimization procedure for *smoke veil* estimation as well as the smoke free image recovery strategy. Finally, in  “Experimental results” section, quantitative and qualitative results are presented and some conclusions are drawn in  “Conclusion” section.

## Related work

To the best of our knowledge, there are only a few recent works related to image processing based laparoscopic desmoking [11–15]. In these papers, the image desmoking problem is considered as a problem similar to dehazing which has been studied for many years in the literature [16, 17]. In such problem, the atmospheric scattering model, presented by Eq. (1), describes the formation of a hazy image through an additive model that is widely used in computer vision [9].

1 $$\begin{aligned} \mathbf {I}(x,y)=\mathbf {J}(x,y)t(x,y)+\mathbf {A}(1-t(x,y)), \end{aligned}$$

where $\mathbf {I}$ is the observed intensity, $\mathbf {J}$ is the scene radiance representing the haze-free image, $\mathbf {A}$ is the global atmospheric light, and *t* is the medium transmission map, considered to decrease exponentially with the light penetration depth (scene depth). A rough mapping between the 3D scene and the projected 2D representation of the transmission map could be expressed as follows:

2 $$\begin{aligned} t(x,y) = e^{-\beta d(x,y)}, \end{aligned}$$

where *d*(*x*, *y*) is the scene depth and $\beta$ is the scattering coefficient [9, 18]. The dehazing process is then reduced to estimate $\mathbf {J}$ by computing the transmission *t* or the depth *d* and the global atmospheric light $\mathbf {A}$.

Based on the above physical model, the recent desmoking methods have been developed by using different tools based on Bayesian inference [11, 12], statistical observation model called refined dark channel prior [13], fusion scheme strategy  [14] and deep learning [15].

Indeed, in [11], the authors formulated a joint desmoking and denoising problem as a Bayesian inference problem based on probabilistic graphical model. This work is then extended in [12] for desmoking, denoising and specularity removal based on learned color probability density functions (PDFs) and undegraded texture priors. In [13], an adapted dark-channel prior combined with histogram equalization method is presented. Directly applying the original dark channel prior dehazing method proposed in [17] would introduce color distortion as a result of the improper assumptions, Tchaka et al. modify the dark channel values by performing a thresholding operation or refining the dark channel values with empirically selected parameters. Then a histogram equalization process is applied to enhance the contrast. A frame by frame analysis shows that the proposed method can reach a better visual quality but a higher MSE (mean square error) compared to the original method presented in [17]. In [14], a visibility-driven fusion defogging framework is proposed. The atmospheric veil is estimated first by a bilateral of bilateral grid (BBG) which is inspired by the work presented in [19]. Then the visibility is recovered by inversing the physical model. A contrast enhancement and luminance fusion scheme are employed to further correct the dark pixels and color distortion. No-reference image quality assessment metrics are used as objective measures to evaluate the obtained results. The results show a promising performance of the method but inefficient in the case of thick and dense smoke. Recently, in [15], a deep learning desmoking method has been proposed. Sabri et al. propose to generate synthetic smoke by Perlin noise [20] which is widely used in computer graphics and embed it to the clear non-smoke images linearly. The synthetic dataset and a dehazing AOD-Net ( All-in-One Dehazing Network) model [21] are then used for transfer learning. This method reaches 20 fps for 512 × 512 color videos but fail in the case of heterogeneous smoke with highly varied spatial density.

While there is few works related to laparoscopic images smoke removal, a similar problem referred to as image dehazing has been studied in the literature [10]. We can simply divide the image dehazing algorithms into two categories: physical model (Eq. 1) based image restoration approaches and non-physical model based approaches.

For the first category, the parameters (e.g. transmission, global atmospheric light) of the physical model are estimated, the restored image is then obtained by inversing the physical model. Many of the image dehazing methods use the atmospheric scattering model and rely on the estimation of the transmission map *t* or the depth map of the images and the global atmospheric light [17, 19, 22]. He et al. propose the dark channel approach based on a statistical observation from outdoor haze-free images: for most of the haze-free natural images, pixel values are very low for at least one channel [17]. The transmission map *t* computed by this prior together with an estimated $\mathbf {A}$ calculated from the detected most haze-opaque region of the image are applied to invert Eq. (1), resulting in a haze free image. This is a well-known efficient approach and lots of recent methods based on it have been proposed [13, 23]. Besides, some deep learning approaches have been proposed to better estimate the physical model’s parameters as no hand-crafted features are required for this kind of approaches. Cai et al. present a CNN (Convolutional Neural Network) DehazeNet to estimate transmission from input hazy images [24]. Later, a end to end AOD-Net is proposed to estimate a new parameter which integrates both *t* and $\mathbf {A}$ [21]. Recently, a new architecture allowing to jointly estimate transmission map, atmospheric light and haze free image has been proposed in [25].

Except the model based approaches, some methods have been developed without estimating transmission or depth maps but by trying for example to improve the contrast [16, 26]. Tan et al. [16] tried to enhance the haze image directly by maximizing the local contrast under an airlight smooth constraint. In [26], a variational contrast enhancement framework for image dehazing with a modified gray-world assumption is proposed. Later in [27], an improved version is presented, where a saturation term is added to the variational cost function aiming to maximize the contrast and saturation together. In [28], Galdran et al. further improved their work by enhancing faraway high-density-fog regions where normally have more fog and preserving nearby low-density-fog regions. It is worth to point out that these methods do not rely on a physical atmospheric model, but try to maximize contrast and saturation. In [29], a multi-scale fusion dehazing method is proposed by deriving a white balance and contrast enhanced inputs. The important features of the two inputs are filtered by luminance, chromaticity and saliency weight maps and then fused by a multi-scale Laplacian and Gaussian pyramid strategy [30] similar to the scheme used in [31] for perceptual contrast enhancement. In [32], a gated fusion network is presented in which three inputs are derived by white balance, contrast enhancement and gamma correction.

Although dehazing and desmoking are similar problems, while haze is related to scene depth, smoke concentration is a local phenomenon which does not depend on the scene depth, but rather depends on the thickness of smoke. Moreover, in laparoscopic images, the light source is provided from the instrument which is not evenly distributed, and the organ surface is not a Lambertian surface. These properties violate the assumptions underlying Eq. (1), which makes it inappropriate to apply directly to laparoscopic images. Therefore, a robust and efficient desmoking approach is highly desired. In this paper, we propose a smoke removal method based on the physical smoke model. The physical model for smoke laparoscopic images and the proposed method will be presented in the following.

## Physical model of smoke image acquisition

**Fig. 1:**
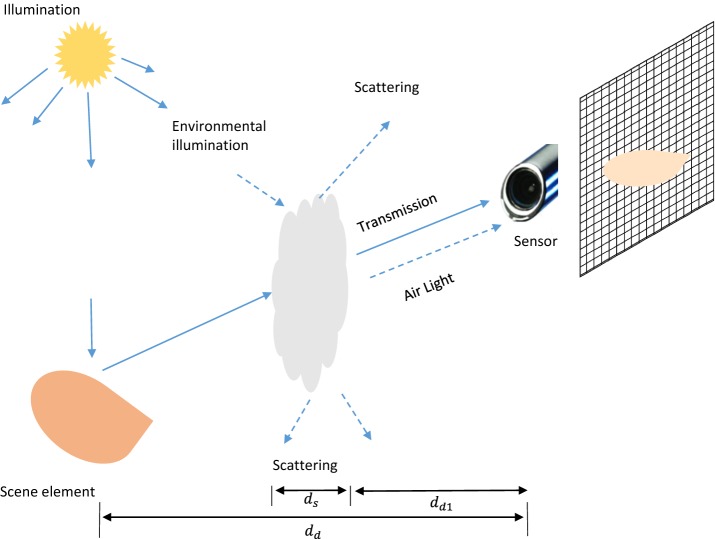
Illustration of smoke image formation

The widely used atmospheric scattering model as proposed in [9] has been applied for natural smoke detection in [33]. Figure 1 illustrates the formation process of smoke images. Although smoke acts as the scattering medium as haze in atmospheric model, it appears from some distance to the camera with a thickness.

For laparoscopic surgery, although the light source is from instrument, the environmental illumination is important in the imaging process. Therefore, the overcast sky illumination model is applied as the direct transmission model where the attenuation of light when it travels through the smoke can be represented by:

3 $$\begin{aligned} \mathbf {L}=g\frac{\eta \rho (\lambda ) e^{-\beta (\lambda ) d_{s}}}{d^{2}_{d}}=\mathbf {J}_{s}e^{-\beta (\lambda ) d_{s}}=\mathbf {J}_{s}t_{s}, \end{aligned}$$

where $\lambda$ is the wavelength, *g* denotes the optical settings of the camera, $\eta$ represents the nature of the illumination, $\rho (\lambda )$ counts for the scene element’s aperture and the scene point’s reflectance, $d_{s}$ is the smoke thickness and $d_{d}$ is the distance between the scene element and imaging sensor (depth), $\mathbf {J}_{s}$ can represent the smoke free radiance and $t_{s}$ is the transmission. Following atmospheric model’s definition introduced in [16, 17], $\mathbf {L}$ is defined as *direct attenuation*.

Except the attenuation of the object intensity, airlight caused by scattering of aerosol/smoke increases the object intensity which is regarded to be the main cause of the shift of scene colors  [17]. Especially for dense smoke, this airlight term would dominate intensity of the scene. According to [9], radiance of airlight is expressed as follows:

4 $$\begin{aligned} \begin{aligned} \mathbf {F}&=\int _{d_{d1}}^{d_{d1}+d_{s}}g\eta \beta (\lambda ) e^{-\beta (\lambda )d} dd =ge^{-\beta (\lambda )d_{d1}}\eta (1-e^{-\beta (\lambda ) d_{s}})\\&=\mathbf {A}_{s}(1-e^{-\beta (\lambda ) d_{s}})=\mathbf {A}_{s}(1-t_{s}), \end{aligned} \end{aligned}$$

where $d_{d1}$ is the distance between sensor and the smoke (Fig. 1). The airlight term $\mathbf {F}$ is referred to as *smoke veil*.

Therefore, the observed image signal could be expressed through the following additive smoke model:

5 $$\begin{aligned} \mathbf {I}=\mathbf {L}+\mathbf {F}=\mathbf {J}_{s}t_{s}+\mathbf {A}_{s}(1-t_{s}), \end{aligned}$$

where $\mathbf {I}$ is the degraded smoke image.

While the mathematical equations of this smoke model (Eq. 5) and the atmospheric one (Eq. 1) have similar form, it is important to note the following two main differences:

1. For the atmospheric model in Eq. (1), $\mathbf {A}$ is a constant and represents the global atmospheric light which can be derived from the radiance of an infinite distance object. For the smoke model, $\mathbf {A}_{s}$ depends on the illumination property $\eta$ which is not a constant as a result of the laparoscope light source and the distance $d_{d1}$ where smoke appears. Thus the conventional approaches used to estimate $\mathbf {A}$ can not be used to estimate $\mathbf {A}_{s}$.
2. The transmission *t* in Eq. (1) depends on the depth of the scene while $t_{s}$ in Eq. (5) depends on the smoke thickness. Therefore, depth related transmission estimation approaches are not suitable for the above smoke model.

## Proposed smoke removal approach

As discussed in the previous section, it is a challenging task to estimate $t_{s}$ and $\mathbf {A}_{s}$ as it is a hard ill-posed problem: according to Eq. (5), there is only one known variable $\mathbf {I}$, but are three unknown variables. In order to solve this under-constrained problem, instead of estimating $\mathbf {A}_{s}$ and $t_{s}$ separately, we propose to perform the task into two steps: estimation of the *smoke veil* $\mathbf {F}$, and computation of the smoke free image $\mathbf {J}_{s}$ from *direct attenuation* $\mathbf {L}$. In this section, the above two steps are described and discussed.

### Smoke veil estimation

The additive *smoke veil* is a whitish or grayish veil which mainly is a function of the properties of illumination and smoke thickness. The illumination might be not evenly distributed, but it is smoothly distributed. Although the smoke thickness is not closely correlated with the depth of the scene, if there is large depth jump of the scene, the smoke thickness has a higher possibility to change. This observation leads us to the assumption regarding to the properties of *smoke veil*: *smoke veil* is smoothly distributed except in regions exhibiting high scene depth changes.

As the physical model assumes that the scattering and transmission properties are independent from wavelength. Therefore, for each channel, the same *smoke veil* value is added to the RGB channels. We can conduct our second assumption: *smoke veil*’s RGB channels’ intensity are equal, which means they have low inter-channel differences (the value of differences between RG, GB and BR channels are low). Our task reduces to estimate a *smoke veil* which satisfies those two assumptions.

We formulate the *smoke veil estimation* as an ill-posed inverse problem and solve it by optimizing variational model with the two assumptions described above. More precisely, an energy function is first defined and then minimized (i.e optimized) via an augmented Lagrangian method, as we shall address next.

#### Energy function

Based on the observations that *smoke veil*’s variation is smooth which means it has low contrast and the RGB inter-channel differences are low, we propose to estimate the *smoke veil* by minimizing the following energy function:

6 $$\begin{aligned} E=\frac{\gamma }{2}\left\| \mathbf {F}-\mathbf {I} \right\| ^{2}+\left\| \mathbf {F}_{TV} \right\| _{2}, \end{aligned}$$

where $\mathbf {I}$ is the degraded color image in the RGB color space, $\mathbf {F}$ is the *smoke veil* to be estimated, $\gamma$ is a scalar to adjust weights between the two terms of the equation, and $\left\| \mathbf {F} _{TV}\right\| _{2}$ is an isotropic total variation (TV)-norm which is given by:

7 $$\begin{aligned} \left\| \mathbf {F} _{TV}\right\| _{2}=\sum _{i} \sqrt{\theta _{x}^2[\mathbf {D}_{x}\mathbf {F}]_{i}^{2}+\theta _{y}^2[\mathbf {D}_{y}\mathbf {F}]_{i}^{2}+\theta _{c}^2[\mathbf {D}_{c}\mathbf {F}]_{i}^{2}}, \end{aligned}$$

where *i* denotes the pixel’s index, $\theta _{x}$, $\theta _{y}$, $\theta _{c}$ are three scalar parameters to balance the weights between the gradient of the color image and the inter-channel differences, and $\mathbf {D}_{x}$, $\mathbf {D}_{y}$, $\mathbf {D}_{c}$ are the forward differential operators along the three dimensions which are computed as:

8 $$\begin{array}{*{20}l} {{\mathbf{D}}_{x} {\mathbf{F}} = {\mathbf{F}}(x + 1,y,c) - {\mathbf{F}}(x,y,c),} \\ {{\mathbf{D}}_{y} {\mathbf{F}} = {\mathbf{F}}(x,y + 1,c) - {\mathbf{F}}(x,y,c),} \\ {{\mathbf{D}}_{c} {\mathbf{F}} = {\mathbf{F}}(x,y,c + 1) - {\mathbf{F}}(x,y,c).{\text{ }}} \\ \end{array}$$

Note that (*x*, *y*, *c*) represents the pixel coordinates of the color image with horizontal and vertical directions (*x*, *y*) and channel direction *c*. Using matrix-vector notation, $[\mathbf {D}_{d}\mathbf {F}]_i$, with $d \in \{x,y,c\}$, denotes the *i*-th component of the one dimensional vector obtained from $\mathbf {D}_{d}\mathbf {F}$.

The first term in Eq. (6) is the data fidelity term which aims to keep the similarity between the estimated *smoke veil* and the input degraded image. In our case, the parameter $\gamma$ is set to a small value to enforce $\mathbf {F}$ to be consistent with $\mathbf {I}$ only for large scale structure part, for example the large depth jump part. As shown in Fig. 3b, when there is depth jump, the corresponding estimated *smoke veil* is not smooth in these regions.

The second term is the regularization term which imposes our assumptions as constraints on the image. TV norm is chosen due to its convexity and edges preserving ability [34]. The TV term would emphasize more when $\gamma$ is small. This term enforces $\mathbf {F}$ to fulfill our assumptions: smoothness or low contrast (low derivative value of $\mathbf {F}$ with respect to variable *x* and *y*) and low inter-channel differences (low derivative value of $\mathbf {F}$ with respect to variable *c*).

#### Optimization method

The nondifferentiability of TV makes the optimization problem of Eq. (6) difficult to solve. The variant of the standard augmented Lagrangian method ADMM (alternating direction method of multipliers) shows its efficiency to solve TV problem [35–37]. Therefore, in this part, the energy function minimization problem is solved by applying the augmented Lagrangian method proposed in [37]. While the latter has been considered in the context of video restoration, it could be easily exploited in our optimization problem since the inter-channel differences can be seen as the temporal variation in videos. To this end, our energy function, given by Eq. (6), is split by introducing an intermediate new variable $\mathbf u$ [37]:

9 $$\begin{array}{*{20}l} {\min _{{\mathbf{F}}} \quad \frac{\gamma }{2}\left\| {{\mathbf{F}} - {\mathbf{I}}} \right\|^{2} + \left\| {\mathbf{u}} \right\|_{2} ,} \\ {s.t.\quad {\mathbf{F}}_{{TV}} - {\mathbf{u}} = 0} \\ \end{array}$$

Following the formalism in [38], the augmented Lagrangian for Eq. (9) is:

10 $$\begin{aligned} L_{\rho }(\mathbf {F},\mathbf {u},\mathbf {y})=\frac{\gamma }{2}\left\| \mathbf {F}-\mathbf {I} \right\| ^{2}+\left\| \mathbf {u} \right\| _{2}+\mathbf {r}^{T}(\mathbf {F}_{TV}-\mathbf {u})+\frac{\rho }{2}\left\| \mathbf {F} _{TV}- \mathbf {u}\right\| ^{2}, \end{aligned}$$

where $\rho$ is a non-negative constant called penalty parameter and $\mathbf {r}=[\mathbf {r}_{x}^{\top },\mathbf {r}_{y}^{\top },\mathbf {r}_{c}^{\top }]^{\top }$ is the Lagrange multipliers vector and $\mathbf {u}=[\mathbf {u}_{x}^{\top },\mathbf {u}_{y}^{\top },\mathbf {u}_{c}^{\top }]^{\top }$. Then, the alternating direction method of Multipliers (ADMM) [38] consists of the following minimization sub-problems:

11 $$\begin{aligned} \begin{aligned} \mathbf F ^{k+1}:=\,&{{\mathrm{argmin}}}_\mathbf{F } L_{\rho }(\mathbf F ,\mathbf u ^{k},\mathbf r ^{k}),\\ =\,&{{\mathrm{argmin}}}_\mathbf{F } \frac{\lambda }{2}\left\| \mathbf {F}-\mathbf {I} \right\| ^{2}+(\mathbf {r}^{k})^{\top }(\mathbf {F}_{TV}-\mathbf {u}^{k})+\frac{\rho }{2}\left\| \mathbf {F} _{TV}- \mathbf {u}^{k}\right\| ^{2}, \\ \mathbf u ^{k+1}:=\,&{{\mathrm{argmin}}}_\mathbf{u } L_{\rho }(\mathbf F ^{k+1},\mathbf u ,\mathbf r ^{k}),\\ =\,&{{\mathrm{argmin}}}_\mathbf{u } \left\| \mathbf {u} \right\| _{2}+ (\mathbf {r}^{k})^{\top }(\mathbf {F}_{TV}^{k+1}-\mathbf {u})+\frac{\rho }{2}\left\| \mathbf {F} _{TV}^{k+1}- \mathbf {u}\right\| ^{2}, \\ \mathbf r ^{k+1}:=\,&\mathbf r ^{k}+\rho (\mathbf F _{TV}^{k+1}-\mathbf u ^{k+1}). \end{aligned} \end{aligned}$$

By introducing the operator $\mathbf D =[\theta _{x}{} \mathbf D _{x}^{\top },\theta _{y}{} \mathbf D _{y}^{\top },\theta _{c}{} \mathbf D _{c}^{\top }]^{\top }$, the $\mathbf F$-minimization subproblem leads to the following solution according to [37]:

12 $$\begin{aligned} \mathbf {F}=\mathcal {F}^{-1}\left[\frac{\mathcal {F}[\gamma \mathbf {I}+\rho \mathbf {D}^{\top }\mathbf {u}-\mathbf {D}^{\top }\mathbf {r}]}{\gamma +\rho \left(\left| \theta _{x}\mathcal {F}[\mathbf D _{x}]\right| ^{2}+\left| \theta _{y}\mathcal {F}[\mathbf D _{y}]\right| ^{2}+\left| \theta _{c}\mathcal {F}[\mathbf D _{c}]\right| ^{2} \right)}\right], \end{aligned}$$

where $\mathcal {F}$ is the Fourier transform operator. Then, the $\mathbf u$ minimization subproblem results in:

13 $$\begin{aligned} \mathbf {u}_{x}=\text{ max }\left\{ \mathbf {v}-\frac{1}{\rho },0 \right\} \cdot \frac{\mathbf {v}_{x}}{\mathbf {v}}, \end{aligned}$$

where $\mathbf {v}_{x}=\theta _{x} \mathbf D _{x}{} \mathbf F +(\frac{1}{\rho })\mathbf r _{x}$. Similar definition is applied to $\mathbf v _{y}$, $\mathbf v _{c}$, and $\mathbf v =\text{ max } \{ \sqrt{\left| \mathbf v _{x}\right| ^{2}+\left| \mathbf v _{y}\right| ^{2} +\left| \mathbf v _{c}\right| ^{2}}, \epsilon \}$ with $\epsilon$ a small constant. In a similar way, $\mathbf u _{y}$ and $\mathbf u _{c}$ are determined to obtain the vector $\mathbf {u}$. More details about these solutions can be found in [37].

### Smoke free image recovery

**Fig. 2:**
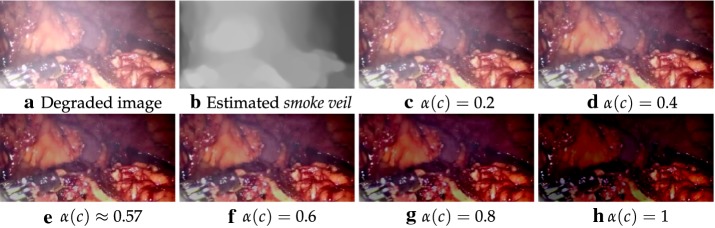
Recovering images using different $\alpha$ values. **a** Degraded image. **b** Estimated *smoke veil*. **c** $\alpha (c)=0.2$. **d** $\alpha (c)=0.4$. **e** $\alpha (c)\approx 0.57$. **f** $\alpha (c)=0.6$. **g** $\alpha (c)=0.8$. **h** $\alpha (c)=1$

After the estimation of global smoke $\mathbf F$, according to Eq. (5), the *direct attenuation* $\mathbf L$ is then calculated as:

14 $$\begin{aligned} \mathbf {L}(x,y,c)=\mathbf {I}(x,y,c)-\alpha (c) \cdot \mathbf {F}(x,y,c), \end{aligned}$$

where $c\in \{R,G,B\}$ indicates the color channel, and $\alpha$ controls how much *smoke veil* is deducted from the input image. A higher value would result in less smoke left in the *direct attenuation*, but would cause darker pixel values as shown in Fig. 2. The latter illustrates the restored smoke free images obtained by different $\alpha$ values. The perceptual smoke density reaches lower levels when $\alpha (c)=0.8$ and $\alpha (c)=1$. However, it leads to much distortion and many pixels become quite dark as shown in Fig. 2g, h.

**Fig. 3:**
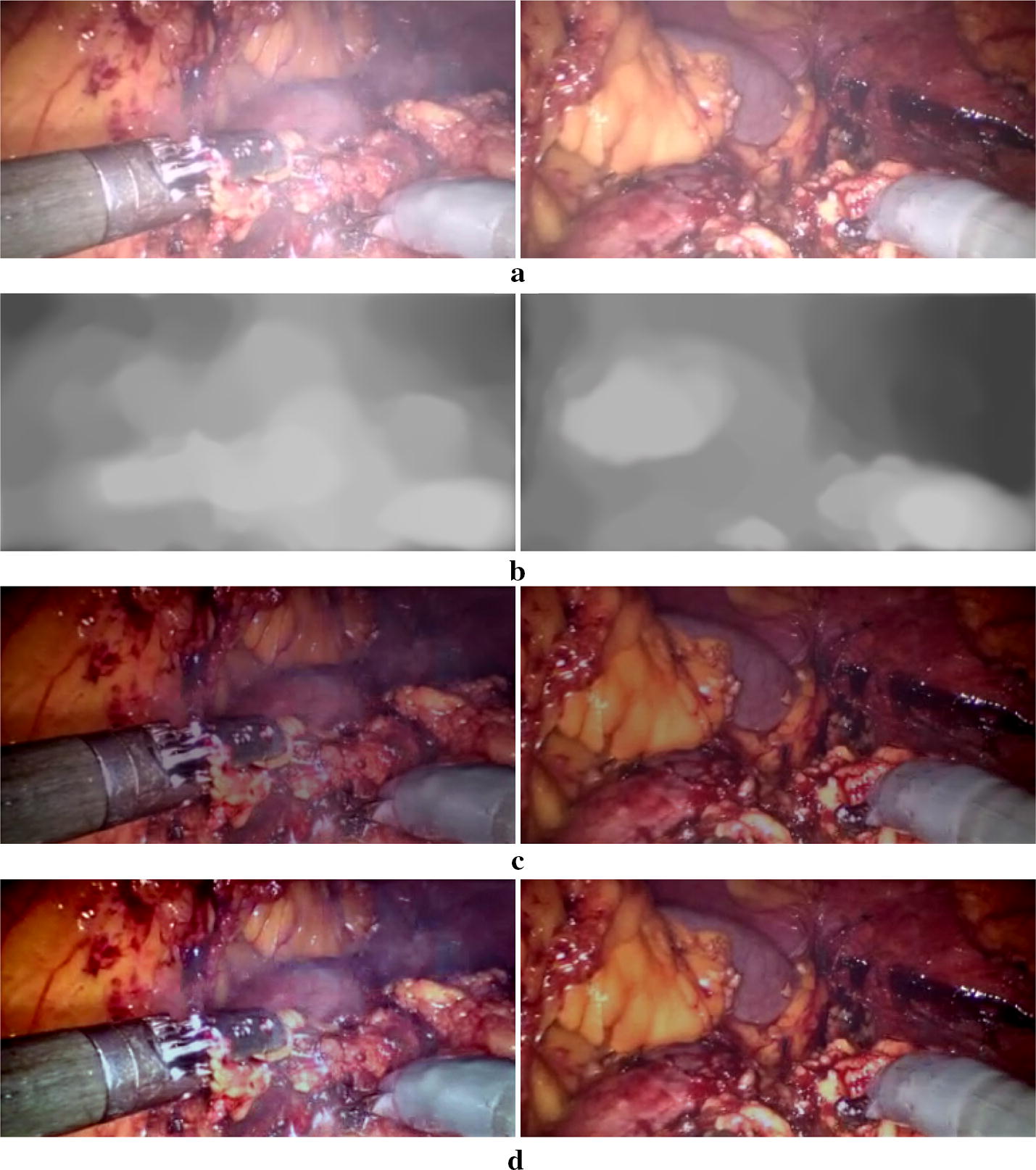
Illustration of the desmoking procedures. **a** The original degraded images. **b** Estimated *smoke veil*. **c** Estimated *direct attenuation*. **d** Final smoke free images

One of the main variables for *smoke veil* is smoke thickness. Higher *smoke veil* values means there is heavier smoke in the image, and so, a higher $\alpha$ value is required. Therefore, $\alpha$ can be simply set to the mean values of the estimated *smoke veil* over the RGB channels. For the example illustrated in Fig. 2, the calculated $\alpha$ values are 0.5747 for R channel, 0.5745 for G channel and 0.5745 for B channel. The estimated smoke free image is displayed in Fig. 2e which shows good visual quality.

Figure 3b illustrates the estimated *smoke veil*. When this part is eliminated from the original degraded image (Fig. 3a), we obtain the *direct attenuation* which is a dim smoke free image as shown in Fig. 3c. From Eq. (3), the object radiance is attenuated exponentially with the thickness of smoke. In laparoscopic surgery, although the smoke thickness changes, the depth range of the scene is limit, which means there would not be large jump for the values of smoke thickness $d_{s}$, and so the variation range of $t_{s}$ becomes small. Therefore, instead of trying to estimate the actual values of smoke thickness or $t_{s}$ which is a challenging task, we apply linear transformation to linearly map the R, G, B channels’ values of $\mathbf L$ (Fig. 3c) to [0; 255] yielding the final smoke free image $\mathbf {J}_{s}$ (Fig. 3d).

## Experimental results

In this section, we present the experimental results to assess the performance of our proposed method. The employed datasets are firstly introduced. Then, a performance analysis of the proposed method is presented based on quantitative and qualitative results. The quantitative evaluation is achieved through the use of no-reference and reduced-reference IQA (image quality assessment) metrics for two real degraded laparoscopic image datasets: *Dataset1* and *Dataset2*, while full-reference IQA metrics are used for a *synthetic dataset*.

In vivo procedure datasets [39, 40], taken from Hamlyn Centre Laparoscopic/Endoscopic Video Dataset Page [41], are used for validation. Real smoked images are selected manually from the original datasets. *Dataset1* has 96 smoked images and *Dataset2* contains 4031 images. Besides, as the ground truth information for a smoke laparoscopic image is not available, we propose to evaluate the method on synthetic data. The latter is created by the method presented in  [15]. According to  [15], Perlin noise is employed to generate synthetic smoke, then the smoke is linearly embedded to the manually selected ground truth smoke free images. Thus, 100 test images are obtained and referred to as *synthetic dataset*.

In order to show the benefits of the proposed method, we will compare it to the following recent ones [13, 17, 27, 28]. This selection of comparison approaches is based on the suitability for desmoking task and the availability of the source code. The first one is the atmospheric model based image dehazing method with dark channel prior [17]. This method will be designated by DCP. It is important to note here that similar approach has been investigated in [13] to remove smoke for laparoscopic images by adding thresholding or refining steps. In the following, this method is denoted by R-DCP. It should be noted that this method has been considered in the qualitative evaluation part. However, it has not been considered in the quantitative evaluation part because of its sensitivity to different parameters which should be empirically selected for input smoked images of the large experimental datasets. The third one, which will be denoted by E-VAR, corresponds to an enhanced variational approach developed in [27]. Finally, the fourth one, designated by F-VAR, is a fusion-based variational technique [28]. E-VAR and F-VAR rely on a mild physical constraint, which are more suitable for desmoking task.

### Influence of the parameters

**Fig. 4:**
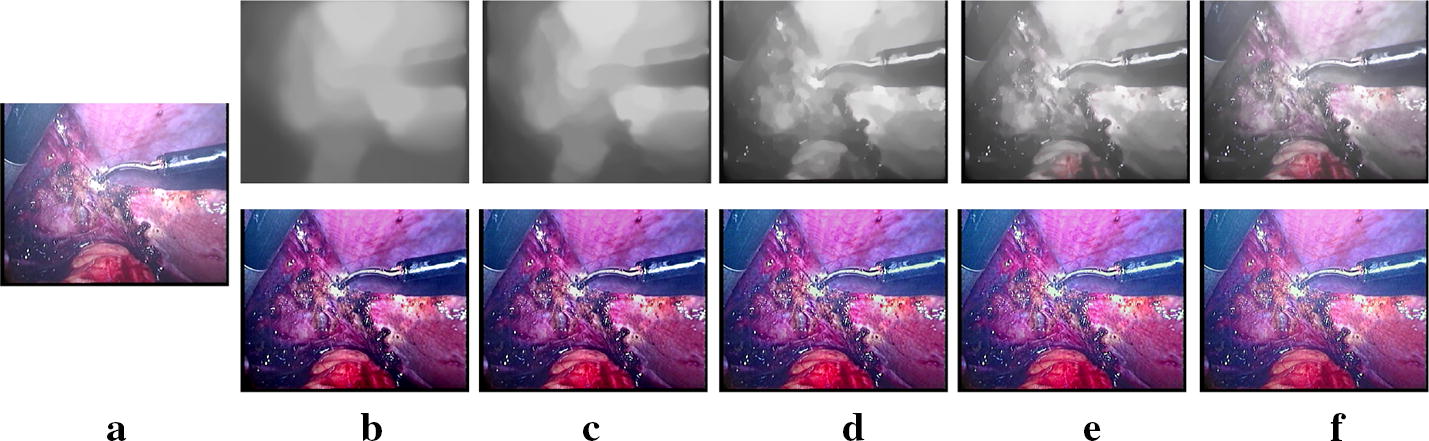
Estimated *smoke veil* and smoke free images using different $\gamma$ values. **a** One original image from *Dataset1*. **b** $\gamma =0.5$. **c** $\gamma =1$. **d** $\gamma =5$. **e** $\gamma =10$. **f** $\gamma =20$

The first key parameter in our smoke removal approach is the regularization parameter $\gamma$ which represents the trade-off between the total variation term and the least square error term defined in Eq. (6). Figure 4 shows the estimated *smoke veil* with different $\gamma$ settings. A smaller value gives a smoother estimated *smoke veil* (Fig. 4b, c), a higher value would force the *smoke veil* more similar to the original image (Fig. 4d–f). Therefore, a smaller $\gamma$ should be chosen as discussed in “Energy function” section. In our experiments, this parameter is set to 1.

Another important parameter in our approach is the $\alpha$ value used in Eq. (14). Let us recall that the effect of this parameter on recovering the smoke free image which has been shown in Fig. 2 and discussed in  “Smoke free image recovery” section. Indeed, compared to the case $\alpha (c)=1$, the obtained results show the benefits of weighting the *smoke veil* during the computation of the *direct attenuation* part. Following this analysis, we proposed in our experiments to set this parameter to the mean value of the estimated *smoke veil* over the RGB channels.

Regarding the other parameters (which had little influence) , they have been chosen as follows: $\theta _{x}=\theta _{y}=\theta _{c}=1$ for Eq. (7) and $\rho =5$ for Eq. (10).

### Quantitative evaluation on *Dataset1* and *Dataset2*

As the ground-truth information for a smoked laparoscopic image is not available, we propose to employ three no-reference IQA metrics and another reduced-reference one that compares the visibility of edges before and after smoke removal. For the purpose of evaluating the ability of smoke removal, a referenceless Fog Aware Density Evaluator (FADE) is employed which has been used to evaluate the perceptual fog density [42, 43]. A lower FADE value means a lower perceptual fog density. Besides, a just noticeable blur based no-reference objective image sharpness metric (JNBM) [44] is used to evaluate the perceptual sharpness. A higher value means higher perceptual sharpness or lower blurriness. Furthermore, we employ a metric, proposed by Hautière et al. [45], which aims to assess the ability of restoring edges (RE) that are not visible in $\mathbf I$ but are in $\mathbf J _{s}$ (obtained after smoke removal). A higher RE value means a better edge restoration. Finally, in order to measure the global image contrast, we proposed to use Mutual Information based Contrast Measure (MICM) recommended in [46]. A lower MICM value indicates higher and better contrast in the images.

**Fig. 5:**
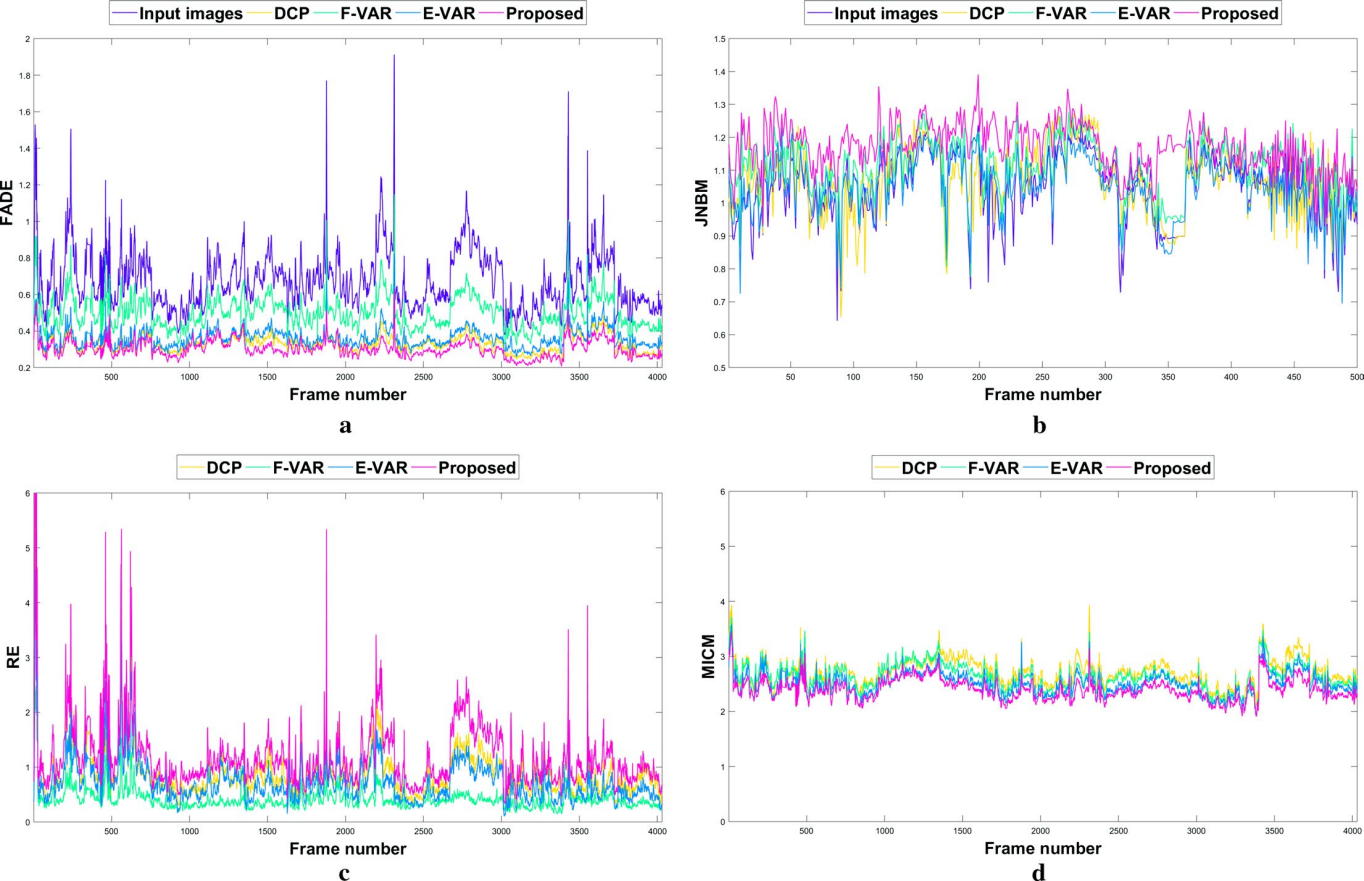
Plotted metrics scores for *Dataset2*. **a** FADE [42]. **b** JNBM [44]. **c** RE [45]. **d** MICM [46]. Note that, for the JNBM, only 500 frames are plotted to provide better illustration

Table 1 shows mean values of the metrics scores of the different approaches for *Dataset1* and *Dataset2*. Figure 5 illustrates the scores of the four metrics on *Dataset2*. All the four metrics show better scores for our approach. In terms of FADE metric, the DCP method removes smoke well. However, it scarifies the perceptual quality as shown in Fig. 7b as a result of the unsuitable underlying constants for desmoking purpose. E-VAR removes more smoke than F-VAR. F-VAR’s results indicate that there are still high smoke density in the images. Our proposed method’s smoke density is the lowest. The proposed approach removes the smooth smoke component of the image resulting in a contrast enhanced image, which has the best scores for JNBM, RE and MICM.

**Table 1:** Quantitative evaluation results for *Dataset1* and *Dataset2*

	Dataset1				Dataset2			
	FADE [42]	JNBM [44]	RE [45]	MICM [46]	FADE [42]	JNBM [44]	RE [45]	MICM [46]
Input images	0.40	1.42	NA	2.62	0.67	1.03	NA	2.85
DCP [17]	0.27	1.57	0.38	2.28	0.33	1.06	0.88	2.72
F-VAR [28]	0.43	1.62	0.12	2.50	0.50	1.09	0.41	2.63
E-VAR [27]	0.35	1.50	0.24	2.13	0.36	1.05	0.73	2.50
Proposed	0.23	1.77	0.39	2.02	0.30	1.16	1.19	2.40

### Quantitative evaluation on *synthetic dataset*

For *synthetic dataset*, full-reference metrics PSNR (peak signal-to-noise ratio) and SSIM (structural similarity index metric) which are widely used in quantitative evaluation of dehazing approaches [21, 25, 32] are used. Besides, MAD (Most Apparent Distortion) which is recommended in [47] is also applied for validation.

Table 2 shows the mean values of the smoke removal results on *synthetic dataset*. Our proposed method outperforms the others in term of all the three full reference image quality metrics.

**Table 2:** Quantitative evaluation results for *synthetic dataset*

	Full-reference IQA metrics		
	PSNR	SSIM	MAD [45]
Input images	18.88	0.81	101.67
DCP [17]	19.18	0.82	116.45
F-VAR [28]	20.16	0.83	100.14
E-VAR [27]	18.99	0.80	117.70
Proposed	20.46	0.86	99.90

### Qualitative visual results

**Fig. 6:**
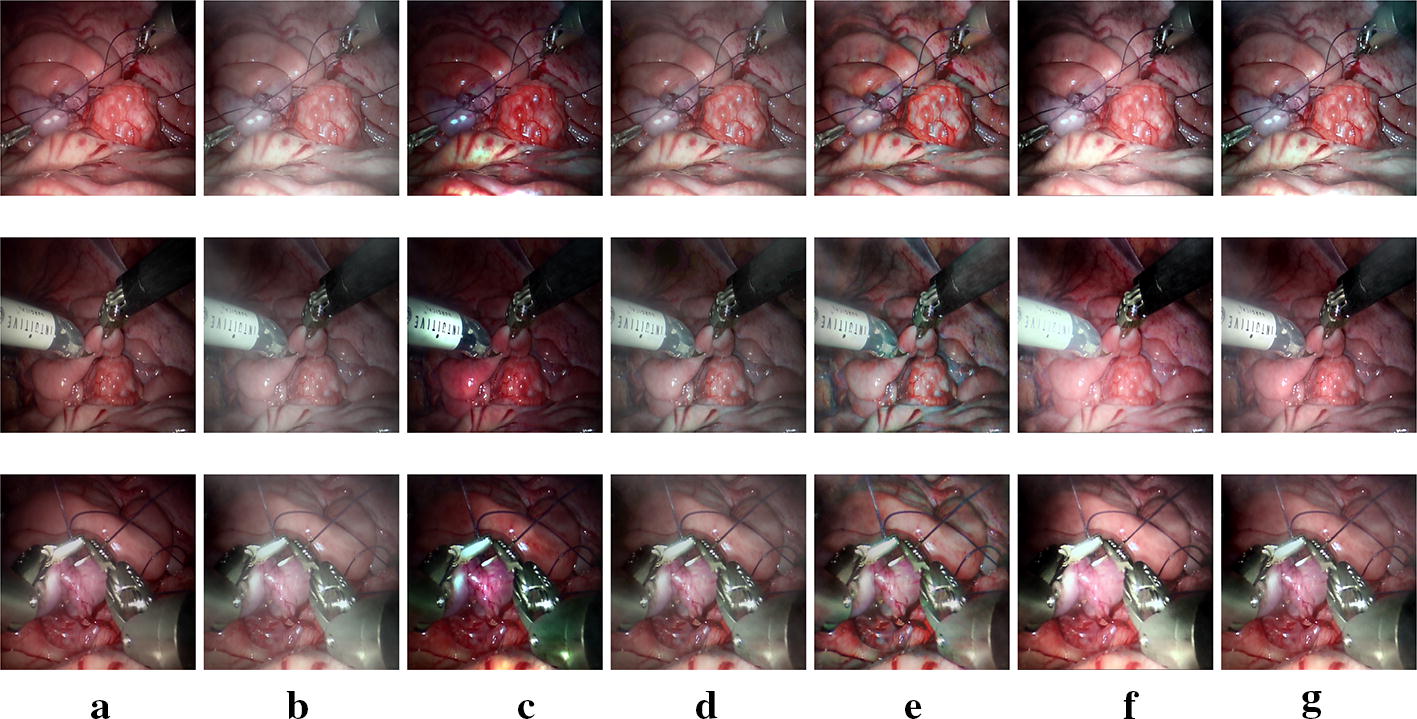
Subjective results for *synthetic dataset*. **a** Smoke free images. **b** Input synthetic smoke images and the obtained desmoked ones using: **c** DCP [17], **d** F-VAR [28], **e** E-VAR [27], **f** R-DCP [13], and **g** proposed method

**Fig. 7:**
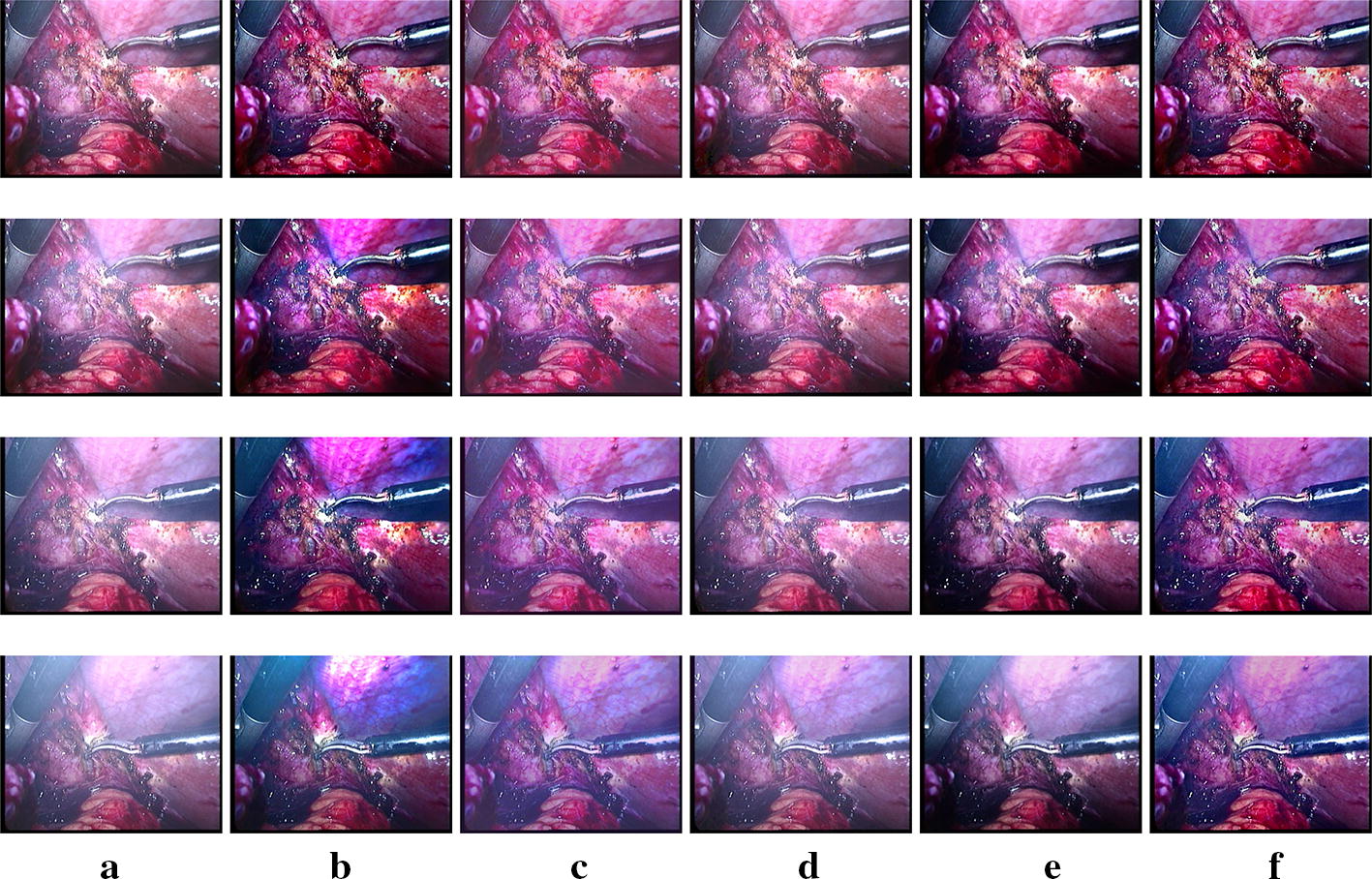
Subjective results for *Dataset1*. **a** Input smoke laparoscopic images and the obtained desmoked ones using: **b** DCP [17], **c** E-VAR [27], **d** F-VAR [28], **e** R-DCP [13], and **f** proposed method

**Fig. 8:**
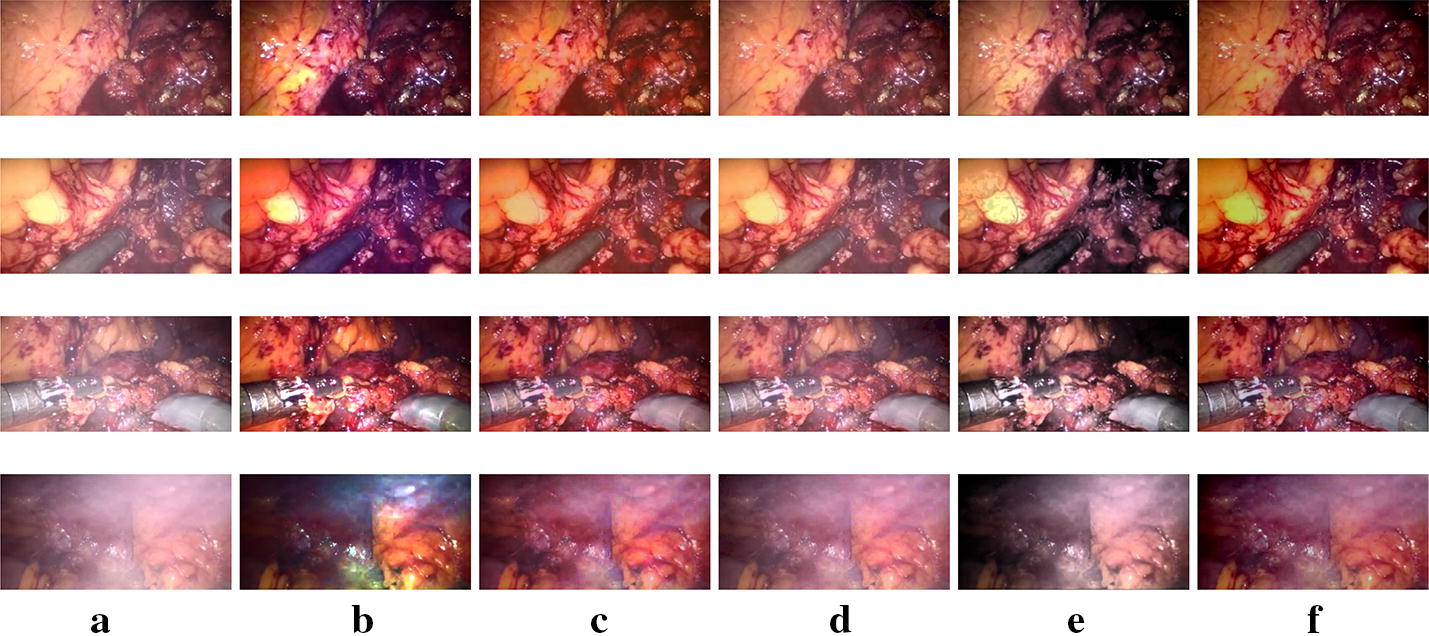
Subjective results for *Dataset2*. **a** Input smoke laparoscopic images and the obtained desmoked ones using: **b** DCP [17], **c** E-VAR [27], **d** F-VAR [28], **e** R-DCP [13], and **f** proposed method

Since there are no gold standards developed for evaluating desmoke images quantitatively, we evaluate the different methods subjectively. Figure 6 shows results from *synthetic dataset*. Figures 7, 8 show results with different smoke density images from *Dataset1* and *Dataset2*.

Figure 6 illustrates examples of the ground truth smoke free images, the synthetic degraded images and the desmoked images from different methods. DCP and E-VAR over enhance the edges. Besides, although DCP and E-VAR show a good smoke remove ability, they both cause color shift. R-DCP shows a fine result, but it causes some blur, for example the instrument part of second row of Fig. 6f. The result obtained by our proposed method shows more pleasant perceptual images.

In Fig. 7, the original images contain low (first row) , moderate and not smooth (second row), moderate and smooth (third row ) and high (last row) smoke density. In Fig. 8, low (first row), moderate (second and third rows), heavy and not smooth (last row) smoke density situations are shown. Our proposed method can remove the smoke well with a pleasant perceptual visual quality except the not smooth ones. DCP removes smoke well but sometimes leads to severe color shift. E-VAR shows a fine result but with over enhanced effect and slightly worse smoke removal compared to our proposed method and the DCP one. F-VAR fails to remove smoke in some images. R-DCP fails to remove the smoke properly for moderate and heavy smoke images and it lacks robust performance when we compare the results from different datasets (Figs. 7e,  8e). Our proposed method achieves well results except the last row of Fig. 8, as dense and heterogeneous smoke violate the underlying assumptions for the physical model. All the methods can not handle dense and heterogeneous smoke well, as shown in the second row of Fig. 7 and the last row of Fig. 8.

Therefore, all the obtained results confirm the benefits of the proposed desmoking method for laparoscopic images.

## Conclusion

In this paper, a variational based desmoking method is presented. The aim is to remove the smoke from the scene, which allows to improve the image guided surgery condition as well as the surgeons’ visibility. Instead of estimating transmission and global atmospheric light as performed in natural image dehazing methods, we estimate the *smoke veil* by a variational method and then estimate the smoke free image based on a simple intensity linear transformation. Quantitative validation on real smoked laparoscopic datasets and synthetic dataset as well as qualitative evaluations are performed. The obtained results show that the proposed approach reduces the smoke effectively while preserving the important perceptual information of the image. A further work related to the acceleration of the proposed method using a GPU implementation is ongoing. Moreover, future research work could be further investigated to improve the smoke free image recovery step by resorting to a fusion strategy and considering temporal information to overcome heavy and heterogeneous smoke images.
